# Toxicity of ZnO and TiO_2_ to *Escherichia coli* cells

**DOI:** 10.1038/srep35243

**Published:** 2016-10-12

**Authors:** Yu Hang Leung, Xiaoying Xu, Angel P. Y. Ma, Fangzhou Liu, Alan M. C. Ng, Zhiyong Shen, Lee A. Gethings, Mu Yao Guo, Aleksandra B. Djurišić, Patrick K. H. Lee, Hung Kay Lee, Wai Kin Chan, Frederick C. C. Leung

**Affiliations:** 1 Dept. of Physics, Univ. of Hong Kong, Pokfulam Road, Hong Kong; 2 School of Energy and Environment, City University of Hong Kong, Kowloon Tong, Hong Kong; 3 School of Biological Sciences, Univ. of Hong Kong, Pokfulam Road, Hong Kong; 4 Dept. of Physics, South University of Science and Technology of China, Shenzhen, China; 5 Pharmaceutical and Life Sciences Division, Waters Corporation, Manchester, UK; 6 Dept. of Chemistry, The Chinese University of Hong Kong, Shatin, New Territories, Hong Kong; 7 Dept. of Chemistry, Univ. of Hong Kong, Pokfulam Road, Hong Kong.

## Abstract

We performed a comprehensive investigation of the toxicity of ZnO and TiO_2_ nanoparticles using *Escherichia coli* as a model organism. Both materials are wide band gap n-type semiconductors and they can interact with lipopolysaccharide molecules present in the outer membrane of *E. coli*, as well as produce reactive oxygen species (ROS) under UV illumination. Despite the similarities in their properties, the response of the bacteria to the two nanomaterials was fundamentally different. When the ROS generation is observed, the toxicity of nanomaterial is commonly attributed to oxidative stress and cell membrane damage caused by lipid peroxidation. However, we found that significant toxicity does not necessarily correlate with up-regulation of ROS-related proteins. TiO_2_ exhibited significant antibacterial activity, but the protein expression profile of bacteria exposed to TiO_2_ was different compared to H_2_O_2_ and the ROS-related proteins were not strongly expressed. On the other hand, ZnO exhibited lower antibacterial activity compared to TiO_2_, and the bacterial response involved up-regulating ROS-related proteins similar to the bacterial response to the exposure to H_2_O_2_. Reasons for the observed differences in toxicity and bacterial response to the two metal oxides are discussed.

There is increasing interest in nanomaterials exhibiting antibacterial activity due to increasing antibiotic resistance^1^. A number of nanomaterials is reported to exhibit significant toxicity and/or antibacterial activity^1^,^2^,^3^,^4^,^5^,^6^,^7^,^8^,^9^,^10^,^11^,^12^,^13^,^14^,^15^,^16^,^17^,^18^,^19^,^20^,^21^,^22^,^23^,^24^,^25^,^26^,^27^,^28^,^29^,^30^,^31^,^32^,^33^,^34^,^35^,^36^,^37^,^38^,^39^,^40^,^41^,^42^,^43^,^44^,^45^,^46^,^47^,^48^, including ZnO and TiO_2_^1^. However, there is no consensus on the proposed mechanism of toxicity of nanomaterials despite numerous literature reports. This can be partly due to problems in standard characterization techniques due to the interaction of nanomaterials with different assays, for example thiobarbituric acid reactive substances (TBARS)^2^,^13^,^24^, as well as instability of some fluorescent probes, which can lead to false positive or false negative results^2^,^5^,^6^,^13^,^24^,^29^,^30^. Other factors such as variation in experimental details (in particular dispersion methods)^5^,^6^, medium in which the testing was performed^19^,^33^, as well as variation in nanomaterial properties^34^, can also affect the obtained results and contribute to the differences among the literature reports. In addition, the majority of the studies report nanomaterial characterization by several methods followed by antibacterial activity testing (commonly by one of the following: plate count method, live-dead discrimination assay, inhibition zone method). In contrast, studies directly evaluating the effect of nanomaterial exposure on molecular processes in the cells, such as transcriptomic and proteomic studies of nanomaterial toxicity^12^,^15^,^21^,^24^,^44^,^45^, have been scarce.

Consequently, there is an interest in combining proteomic investigation of antibacterial activity of nanomaterials with comprehensive nanomaterial characterization. Among various nanomaterials exhibiting antibacterial activity and/or toxicity to various organisms and cell lines, ZnO and TiO_2_ are the most commonly studied ones^1^. Nevertheless, the direct comparisons of these two materials have been scarce^9^,^13^,^17^,^48^,^49^. Both materials are known to be good photocatalysts which are capable of inactivation of microorganisms, and they have similar wide band gap in the UV spectral region^7^. Consequently, both materials are capable of producing reactive oxygen species (ROS) under UV illumination^9^. ROS generated can include hydroxyl radicals OH^•^, superoxide ions O_2_^−•^, and singlet oxygen ^1^O_2_^2^,^17^. However, while TiO_2_ exhibits excellent chemical stability, ZnO is stable only in a very narrow range of pH values and it typically releases Zn^2+^ in aqueous solutions^7^. Thus, since both materials produce ROS and only ZnO releases metal ions, the comparison between these two materials is of interest to investigate the commonly proposed mechanisms of toxicity, namely ROS generation^1^,^2^,^4^,^13^,^17^,^22^,^26^,^31^,^32^,^37^,^40^,^41^,^42^,^46^,^47^,^48^ and metal ion release^1^,^4^,^17^,^18^,^19^,^35^.

For both of these mechanisms, contradictory results have been reported in the literature. A number of studies reported that ROS play a significant role in nanomaterial toxicity, in particular under illumination^2^,^4^,^6^,^13^,^17^,^22^,^26^,^31^,^32^,^37^,^40^,^41^,^42^,^46^,^47^,^48^, while others reported no correlation between ROS production and toxicity^10^,^12^,^16^,^21^,^24^,^29^,^30^,^34^,^43^. Similarly, contradictory reports exist on the issue of Zn^2+^ release contribution to toxicity, with some studies attributing the toxicity to zinc ion release^1^,^4^,^17^,^18^,^19^,^35^ while in others no correlation was observed^17^,^30^,^31^,^34^,^37^. Also, in addition to these two commonly proposed mechanisms, other mechanisms such as electron transfer between the nanomaterial and bacteria membrane, resulting in ROS-independent oxidative stress^16^,^27^, as well as phosphate starvation^20^ have been proposed.

Unlike conventional studies of evaluating antibacterial activity and measuring ROS production, followed by proposing the mechanism of activity based on observed correlations (if any), proteomics investigations can provide direct evidence on the toxicity mechanism by detecting changes in protein expression as a reaction of exposure of bacteria to the nanomaterial. The importance of proteomics investigations in conclusive determination of toxicity mechanism was demonstrated recently in a study of the response of *Cupriavidus necator* bacteria to exposure to ZnO nanoparticles and Zn^2+^ ions^44^. It was found that although the toxicity of ZnO nanoparticles and zinc acetate (source of Zn^2+^ ions) was similar based on *in vitro* studies, up-regulation of different proteins was observed upon exposure to ZnO and Zn^2+ ^44. Up-regulation of cell membrane proteins was observed only for exposure to ZnO nanoparticles, indicating that the toxicity mechanism involves membrane disruption which is different from exposure to Zn^2+ ^44. Thus, proteomic studies combined with comprehensive nanomaterial characterization and conventional *in vitro* toxicity testing can offer insight into understanding the nanomaterial-bacteria interaction and the mechanism of toxicity. Therefore, we performed comprehensive study of antibacterial activity of ZnO and TiO_2_ nanoparticles using not only common methods of studying antibacterial activity (cell counts, ROS generation), but also proteomics studies. The mechanism of antibacterial action was also comprehensively investigated including ROS detection, lipid peroxidation detection, phosphate adsorption, interaction between the nanoparticles and bacteria, and proteomics investigation. The tests were conducted under optimal conditions for UV illumination (isotonic, non-light absorbing medium)^33^ on *E. coli* as a model organism. Both materials are capable of ROS generation, while metal ion release occurs only for ZnO. In addition to using proteomics investigations to elucidate cell responses to exposure to nanoparticles, we also examined the effect of varying the test conditions (nanoparticle and bacteria concentrations, while keeping testing medium the same since testing medium was chosen to be optimal). In all tests, no dispersion agents or surfactants were used to eliminate artefacts caused by these agents^6^,^50^,^51^. Consequently, aggregation sizes for different nanoparticle concentrations in test medium were determined, since this is one of the key parameters affecting the toxicity^52^. We found that the obtained results are strongly dependent on both nanoparticle and bacteria concentrations in a nonlinear way (increasing nanoparticle concentration does not necessarily result in the increasing toxicity).

## Results and Discussion

**Figure 1:**
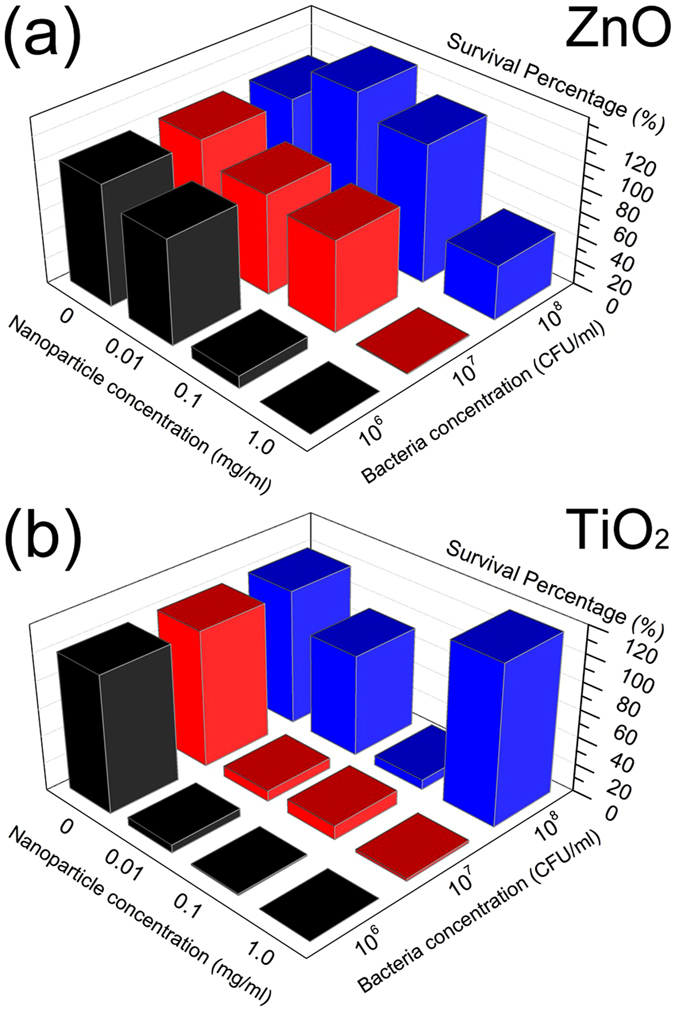
Survival percentages of *E. coli* bacteria after UV illumination for 20 min. For different starting bacterial and nanoparticle concentrations (**a**) ZnO nanoparticles (**b**) TiO_2_ nanoparticles.

The obtained results of antibacterial activity testing for ZnO and TiO_2_ are shown in Fig. 1 (and in Supplementary Information, Tables S1 and S2, respectively). ZnO exhibits significant antibacterial action for the highest concentration (1 mg/ml) for all bacteria concentrations under UV illumination. For the same nanoparticle concentrations, increasing bacteria concentration results in a reduction of antibacterial activity, as expected since there are fewer nanoparticles available for each bacterial cell. ZnO also exhibits significant antibacterial activity in the dark, which is more pronounced for lower bacteria concentrations (10^6^ CFU/ml and 10^7^ CFU/ml). For 10^8^ CFU/ml, similar level of small reduction of colony counts is observed for all three concentrations. This indicates that there is an illumination-independent toxicity mechanism in the case of ZnO.

However, different trends are observed for TiO_2_. Under UV illumination and for the highest bacteria concentration, significant antibacterial action is observed only for 0.1 mg/ml nanoparticle concentration, while the highest nanoparticle concentration (1 mg/ml) exhibits no toxicity. This is entirely different from the results obtained for lower bacteria concentrations, where the highest toxicity is observed for the highest nanoparticle concentration (1 mg/ml). These surprising trends are reproducible (experiments repeated on different days resulted in the same trends). Similar to ZnO, in the dark no concentration dependent toxicity trends were observed for the highest bacteria concentration, while for the lower concentrations there is some effect though significantly smaller than in the case of ZnO. From the characterization of the nanoparticle suspensions, it can be observed that there are significant differences in the aggregation sizes and turbidity depending on the material, while for zeta potential no clear trends could be observed. The differences in turbidity (for the appearance of suspensions, see Supplementary Information, Fig. S1) would affect the penetration of the UV light in the suspension and thus the observed antibacterial activity. It should also be noted that, according to supplier information, the particles have similar nominal sizes (20 nm for ZnO, 15 nm for TiO_2_). Transmission electron microscopy (TEM) examination of the particles revealed that the actual particle sizes are ~30 nm for ZnO and ~10 nm for TiO_2_ (see Supplementary Information, Fig. S2). Nevertheless, individual particle size is not a reliable predictor of an aggregate size in solution, which is dependent on a number of variables, such as the dispersion medium, particle concentration and time^52^. Thus, as shown in Table 1, larger ZnO nanoparticles form smaller aggregates compared to TiO_2_ at low particle concentration (0.01 mg/ml), while TiO_2_ nanoparticles form smaller aggregates compared to ZnO at high particle concentration (1 mg/ml). Furthermore, there is no clear correlation between aggregate size and antibacterial activity.

In addition, differences in turbidity cannot explain the observed differences in antibacterial activity without illumination. Furthermore, no significant titanium release occurs from TiO_2_ in 0.9% *w/*v NaCl solutions^9^, so that antibacterial activity in the absence of illumination cannot be explained by metal ion release. Moreover, TiO_2_ exhibits more prominent antibacterial activity compared to ZnO under UV illumination, while the opposite is the case in the absence of illumination. In our previous work^9^, we have found that ZnO exhibited higher antibacterial activity compared to TiO_2_ for experiments performed on nanoparticle coated slides as opposed to suspensions with different nanoparticle concentrations in this work. This confirms that experimental conditions have critical influence on the obtained results, since we used the same batch of nanoparticles from the same supplier for these experiments. For other comparisons of antibacterial activity of ZnO and TiO_2_ in the literature, the differences in the obtained results^9^,^17^,^48^,^49^ can be attributed not only to the differences in the experimental procedures but also to the differences in nanomaterials used.

**Figure 2:**
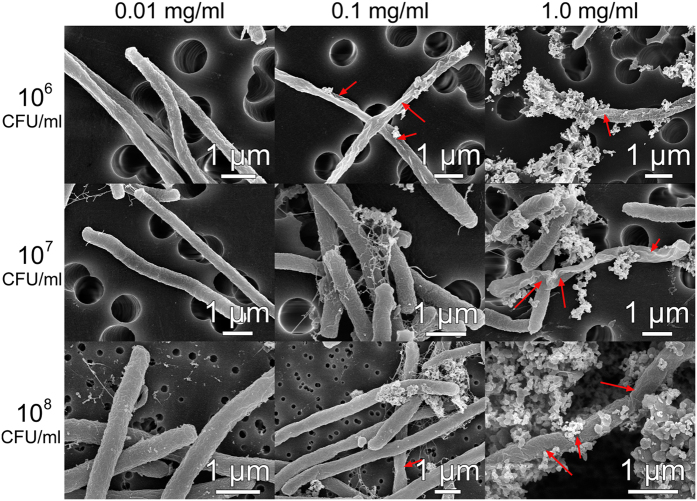
Representative SEM images of *E. coli* bacterial cells after exposure to ZnO nanoparticles with different concentrations (0.01, 0.1 and 1.0 mg/ml). Left-most column indicates the initial bacterial concentrations (10^6^, 10^7^ and 10^8^ CFU/ml).

**Figure 3:**
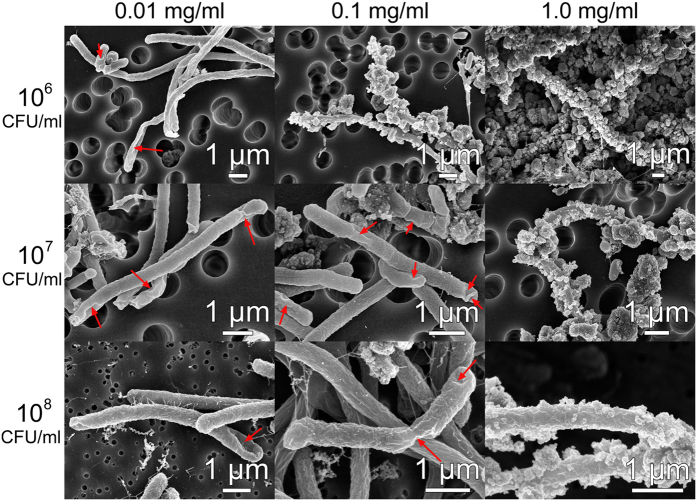
Representative SEM images of *E. coli* bacterial cells after exposure to TiO_2_ nanoparticles with different concentrations (0.01, 0.1 and 1.0 mg/ml). Left-most column indicates the initial bacterial concentrations (10^6^, 10^7^, and 10^8^ CFU/ml).

To further examine mechanisms of antibacterial action, SEM imaging of the bacteria after exposure to nanomaterials was performed. The corresponding representative SEM images are shown in Figs 2 and 3, respectively (see Supplementary Information, Fig. S3 for SEM images of bacteria without exposure to nanomaterials, Fig. S4 for additional images of bacteria exposed to TiO_2_ at a concentration of 0.1 mg/L, and Figs S5 & S6 for additional SEM images of bacteria for all conditions shown in Figs 2 and 3). At low particle concentrations (0.01 mg/ml), fewer nanoparticles and fewer cell damage sites are observed, while for large particle concentration (1 mg/ml) abundant nanoparticles are present, with cell damage more common for ZnO than for TiO_2_ in agreement with antibacterial activity testing. For 0.1 mg/ml, cell damage is more commonly found for TiO_2_ than for ZnO, and more particles are attached to cells for lower bacteria concentrations as expected. Thus, clear cell damage (holes in the cell membrane, indicated by arrows, with bacteria deformation and collapse due to the leakage of contents) is commonly found for samples exhibiting significant toxicity, in agreement with the literature observations^14^,^16^,^23^,^24^,^28^. The cell membrane damage is commonly attributed to the effects of ROS^14^,^26^,^40^,^41^,^47^. However, other mechanisms have also been proposed, and cell membrane damage due to the interaction between the nanoparticles and the cell membrane has been observed in the absence of ROS^24^. Such interaction can result in a damaged molecular structure of phospholipids and consequently cell membrane damage^39^. From the SEM images of the bacteria, we can see that the membrane damage occurs and that there is attachment of nanoparticles to bacteria, but the exact mechanism how damage occurs could not be identified. Some of the large holes in the cell membranes (see Supporting Information, Fig. S6, 10^7^ CFU/ml, 0.1 mg/ml) do not occur in proximity of the attached nanoparticles and thus it is not clear how they formed.

Particle attachment is more commonly observed for TiO_2_ than for ZnO. However, in the case of 1.0 mg/ml of TiO_2_ and bacteria concentration of 10^8^ CFU/ml, clear attachment of the nanoparticles can be observed, but there is no toxicity and no cell membrane damage. Thus, there is no clear correlation between the nanoparticle attachment and the antibacterial action of the nanoparticles. Furthermore, positive zeta potential value is obtained only for 1 mg/ml ZnO suspensions, which indicates that electrostatic interaction between nanoparticles and negatively charged bacteria can play a role in the antibacterial activity only for this particular sample. For samples with negative zeta potentials, the sorption of nanoparticles onto the cell membranes likely occurs by other mechanisms, such as Van der Waals forces, hydrophobic and receptor-ligand interactions^23^,^24^.

**Figure 4:**
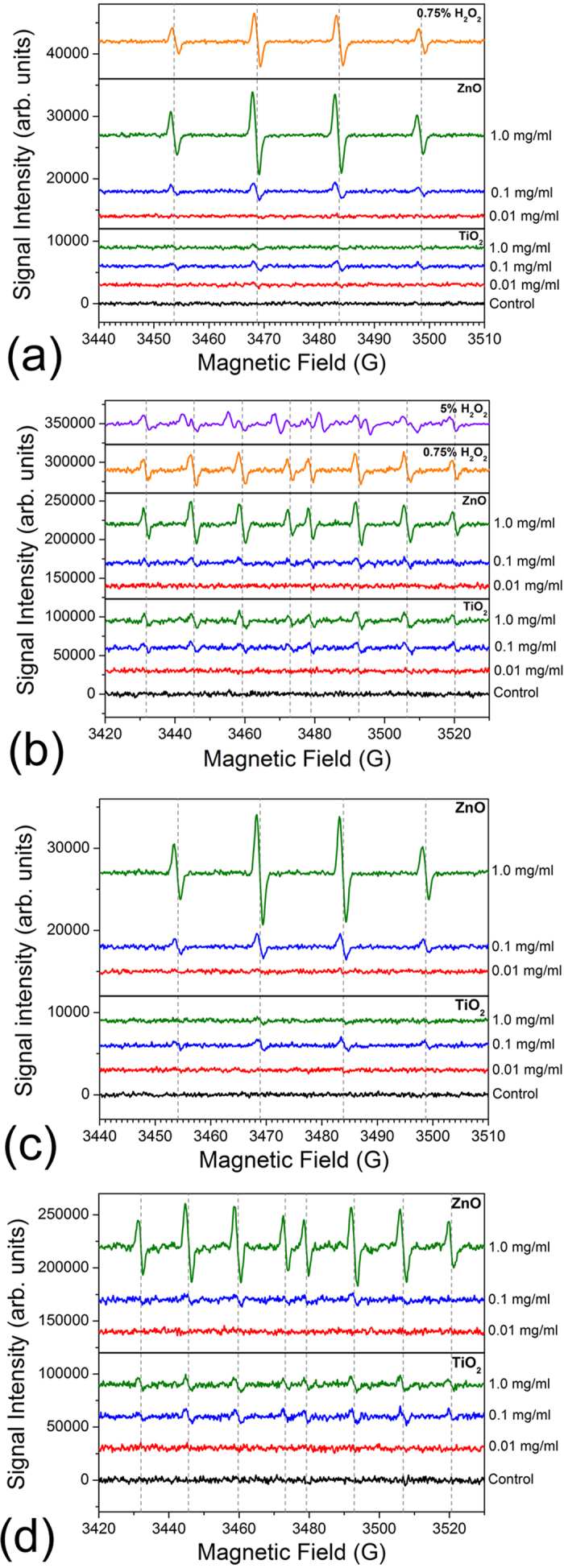
Electron spin resonance (ESR) spectra for ZnO and TiO_2_ nanoparticles with different concentrations (**a**) with DMPO spin trap, without *E. coli* (**b**) DEPMPO spin trap, without *E. coli*. (**c**) DMPO spin trap, with *E. coli* (**d**) DEPMPO spin trap, with *E. coli*. In all cases, *E. coli* concentration was 10^6^ CFU/ml. The spectra of H_2_O_2_ solutions are also shown for comparison.

To examine reasons for the observed cell membrane damage, we determined nanoparticle properties (summarized in Table 1) and examined ROS generation, as shown in Fig. 4. ESR was used for ROS detection as a more reliable technique compared to fluorescent probes^30^,^46^. For both nanomaterials, same trends are observed with and without bacteria. No significant ROS generation was detected for the lowest nanoparticle concentration, likely due to the fact that it was below the detection limit. The dominant ROS type generated is OH^•^ radicals, which have been previously identified as responsible for antibacterial action of TiO_2_^3^,^21^. ZnO tends to produce more ROS compared to TiO_2_. For both materials, ROS production does not appear to be related to the aggregation size in solution (which exhibits nonlinear dependence on the concentration).

The ROS generation was proposed to result in the degradation of the cell membrane^26^, for example by lipid peroxidation^38^,^47^, which leads to the leakage of cellular contents and ultimately cell death^3^. To examine this possibility, TBARS assay was conducted, since it is commonly used for the detection of lipid peroxidation^13^,^24^,^29^,^38^,^41^. Since TBARS assay can lead to artefacts due to interaction with nanomaterial^29^, the samples with nanomaterial without bacteria (with TCA added) were also examined and no significant signal at 532 nm was observed. Obtained results are summarized in Supplementary Information, Table S3. While in some cases (10^8^ CFU/ml, TiO_2_) correlations with the antibacterial activity are observed, this is not true for an entire dataset (for example 10^7^ CFU/ml). On the other hand, linear dependence between the nanoparticle concentrations, Zn^2+^ ion release, and antibacterial activity of ZnO can be observed (higher concentrations result in larger aggregation size, increased Zn^2+^ ion release, and increased antibacterial activity). However, the toxicity could not be attributed to the Zn^2+^ ion release since the highest detected zinc ion concentration did not result in a significant reduction in bacteria survival rates (see Supplementary Information, Table S4), in agreement with other reports in the literature^18^. In addition, ZnO toxicity to *E. coli* is lower than that of TiO_2_. Thus, neither Zn^2+^ ion release nor ROS can explain the observed trends in the antibacterial activity. The studies attributing the toxicity to ROS^2^,^4^,^6^,^13^,^17^,^22^,^26^,^31^,^32^,^37^,^40^,^41^,^42^,^46^,^47^,^48^ typically observed correlation between ROS production and/or lipid peroxidation and cell death using different assays (fluorescent probes, TBARS)^46^,^47^,^48^, while actual verification of proposed mechanism by examining cell response to nanomaterial exposure using proteomics have been scarce. Correlation between ROS production and antibacterial activity could be accidental, or ROS production and resulting oxidative stress could be just one of possible mechanisms of antibacterial activity, with dominant mechanism determined by the properties of specific nanomaterial samples. Thus, other possible hypotheses need to be carefully examined, and proteomics investigations conducted to obtain unambiguous answers to whether oxidative stress is a significant factor in antibacterial activity of these nanomaterials.

To examine other possible mechanisms of antibacterial activity, modification of nanoparticles with phosphate and experiments in the presence of sodium phosphate were performed. The addition of sodium phosphate and phosphate modification have significant effect on suppressing the antibacterial activity of ZnO, but only small effects are observed in the case of TiO_2_ (Table S5 and Fig. S7). For ZnO, large particle aggregates are observed in SEM images, which is likely due to expected formation of zinc phosphate^9^,^19^. For TiO_2_, no significant impairment of the particle attachment is observed, in agreement with only a small change in the level of antibacterial activity but different from previous report^28^. Since various components of the cell wall, such as carbohydrate-related moieties, carboxyl, amide, phosphate and hydroxyl groups can participate in the interactions with nanoparticles^23^,^24^, phosphate modification and the presence of phosphate in the solution likely affect only some of the possible interaction sites. Both ZnO and TiO_2_ with and without phosphate modifications exhibit similar interaction with lipopolysaccharide (LPS). LPS is the main molecule present in the surface layer of the outer membrane of *E. coli* and it is found exclusively in the outer membrane^26^. When nanoparticles are exposed to LPS, followed by rinsing, the attachment of LPS indicates that nanoparticles are likely to interact with and attach to the outer membrane of *E. coli* which mainly consists of LPS molecules. FTIR spectra of nanoparticles exposed to LPS are shown in Fig. S8, Supplementary Information. Clear attachment of LPS can be observed for ZnO since new peaks at ~2921 cm^−1^ and 2855 cm^−1^ corresponding to CH_2_ vibrations can be clearly observed, as well as the peak at ~1232 cm^−1^ corresponding to PO^2−^ vibrations^34^. For TiO_2_, we can observe an appearance of peak at ~1214 cm^−2^ which could possibly due to PO^2−^ vibrations^26^,^34^, but the peaks corresponding to CH_2_ and CH_3_ vibrations cannot be resolved, indicating less strong interaction between LPS and TiO_2_ compared to ZnO, different from antibacterial activity trends. Another difference observed in TiO_2_ is that peak at ~1400 cm^−1^, which likely corresponds to COO- vibrations^26^, disappears both after exposure to LPS and phosphate pre-treatment, indicating a change in the surface adsorbates of TiO_2_.

Next, proteomics investigations were performed to understand the responses at the molecular level. *E. coli* samples exposed to H_2_O_2_ have also been investigated as a positive control for the effects of ROS. H_2_O_2_ concentration was selected to result in a moderate antibacterial activity, between the lowest observed percentages of survival for ZnO and TiO_2_ for 10^8^ CFU/ml (concentration required for preparing samples for proteomics). Furthermore, to examine the effects of cell membrane damage without an external source of ROS, bacteria samples subjected to thermal stress have also been included, and antibacterial activity results and SEM images are shown in Supplementary Information (Tables S6 and S7, Fig. S9). The proteomic responses were analyzed by LC/MS after cells were incubated with nanomaterials for various durations with or without illumination, H_2_O_2_ or thermal stress. In total, 1,586 unique proteins were identified where 649 and 833 proteins showed changes ≥1.5 fold (up- or down-regulated) in the experiments with TiO_2_ and ZnO, respectively. In the H_2_O_2_ and thermal stress experiments, 947 and 513 proteins showed changes ≥1.5 fold, respectively. To identify statistical significant ROS-related and outer membrane proteins, a protein has to have FDR ≤1%, peptide count ≥3, and fold-change ≥2.

**Figure 5:**
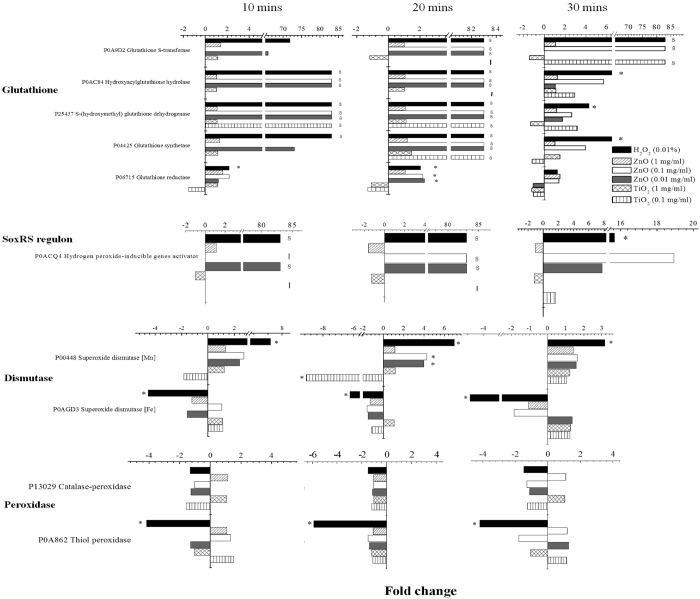
Expression of ROS-related proteins under different conditions. “∞” represents the expression of proteins in the treated cells but no protein was detected in the control. “−” represents no protein was detected in both the treated and control cells. “*”represents genes that were statistically significant (FDR ≤1%, peptide count ≥3, fold-change ≥2). Expression ratios were calculated with the protein abundance measured in the control as the denominator.

**Figure 6:**
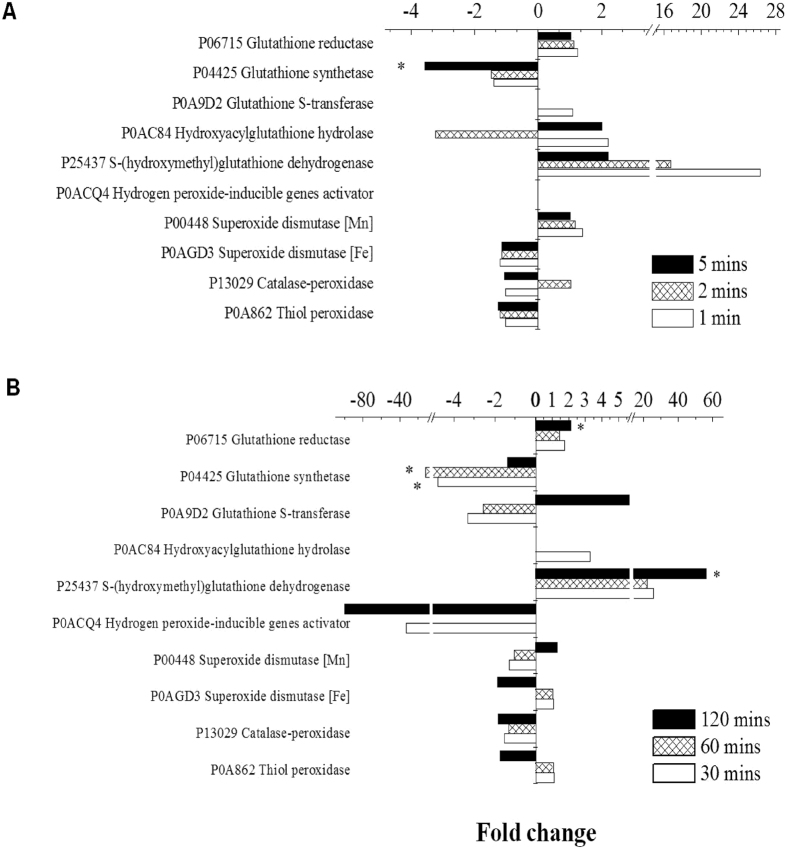
Expression of ROS-related proteins without UV illumination. (**A**) Thermal stress treated cells. (**B**) ZnO (0.1 mg/ml) treated cells. “*” represents genes that were statistically significant (FDR ≤1%, peptide count ≥3, fold-change ≥2). Expression ratios were calculated with the protein abundance measured in the control as the denominator.

ROS are widely reported^28^,^46^,^47^,^48^,^49^,^53^ to be the main cause leading to extensive cell damages at both cellular and molecular levels. Correspondingly, bacteria can express ROS-related proteins such as glutathione-related enzymes, the SoxRS regulon, superoxide dismutases, and peroxidases as part of the mechanisms to defend against oxidative stresses^54^,^55^,^56^,^57^. Since less ROS production could be detected in the cultures exposed to TiO_2_ by ESR spectra and a non-linear correlation was obtained by the lipid peroxidation tests, the underlying bactericidal mechanism between TiO_2_ and ZnO probably differed. When incubated with ZnO at a concentration less than or equal to 0.1 mg/ml, many enzymes related to glutathione and the SoxRS regulon were highly expressed and the peak expression occurred after 20 minutes of incubation, as shown in Fig. 5. In fact, many of the ROS-related proteins were not detected in the controls without ZnO (i.e. on/off responses). The expression profile of ROS-related proteins between ZnO treated samples and the H_2_O_2_ positive control was largely similar, suggesting that ZnO trigged similar oxidative stress as H_2_O_2_ with the involvement of radical species. In both the H_2_O_2_ and ZnO samples, the Fe-superoxide dismutase and peroxidases were mostly down-regulated, but the Mn-superoxide dismutase was up-regulated. In the thermal stress treated cells where no ROS were expected to be generated, ROS-related proteins were not significantly expressed within five minutes of incubation as shown in Fig. 6a. In the ZnO treated cells without UV illumination, significant up-regulation of ROS-related proteins was noted for only two proteins (glutathione reductase and glutathione dehydrogenase) after 120 minutes of incubation (Fig. 6b). In contrast to ZnO and H_2_O_2_, many of the ROS-related proteins could not be identified as significantly up-regulated when cells were incubated with 0.1 mg/ml and 1 mg/ml TiO_2_ regardless of the length of incubation, as shown in Fig. 5. Only the glutathione dehydrogenase (at 10 and 20 minute), glutathione synthetase (at 20 minute) and glutathione S-transferase (at 30 minute) were up-regulated in the samples with 0.1 mg/ml TiO_2_. Given that the overall protein expression profile of TiO_2_ seemed different than H_2_O_2_, this suggests the bactericidal mechanism of TiO_2_ is not strongly coupled to ROS generation. Based on the results that the ROS-related proteins were not strongly expressed in both 0.1 mg/ml and 1.0 mg/ml TiO_2_, it is not obvious what factors contribute to the differences in survival rate (7% vs. ~100%) (Table S2) between the two treatments.

The concentration of oxidants and the duration of incubation are important factors that influence the expression of ROS-related genes. For example, the expression of catalase in *Salmonella typhimurium* was not detected either during the first 15 minutes of incubation or in the presence of exogenous H_2_O_2_ below a concentration of 0.1 μM^58^. Interestingly, in this study, we found that when cells were incubated at the highest ZnO concentration of 1 mg/ml, a low survival rate (44.3%) was observed. Coincidentally, ROS-related proteins were not significantly expressed in these cells (Fig. 5). In contrast, when cells were incubated with a lower concentration of 0.01 or 0.1 mg/ml ZnO, ROS-related proteins were highly up-regulated and a higher survival rate (~100%) was obtained (Fig. 5, Table S1). Hence, there seems to be a correlation between the ability of cells to withstand the antibacterial activity of ZnO and the expression of ROS-related proteins as possible defense mechanisms. Alternatively, at 1 mg/ml of ZnO, cells could have been very rapidly inactivated that ROS-related genes were not expressed by the time the first measurement was made after 10 minutes of incubation. Further experiments are required to resolve the timescale of inactivation for 1 mg/ml of ZnO.

Electron microscopy images have indicated that for TiO_2_-driven bactericidal activity (except for 1 mg/ml) direct contact resulted in physical damages of the cell membrane. Consistent with this observation, cell membrane proteins were mostly down-regulated in the samples incubated with TiO_2_ after 10 minutes. The expression of outer membrane protein A (OmpA), OmpC, OmpF, and OmpW showed a down-regulation trend of about two to seven folds at this time point, but little changes were observed at 20 and 30 minutes (Table S8). For the ZnO (0.1 and 0.01 mg/ml) samples, except for OmpA, a number of outer membrane proteins were up-regulated. Similarly, for the H_2_O_2_ samples, the OmpA was significantly down-regulated and OmpW was significantly up-regulated (Table S8). For both the TiO_2_ and ZnO at 1 mg/ml experiments, the outer membrane proteins showed little changes in expression (Table S9). These results suggest that outer membrane proteins could be a target for TiO_2_, which is consistent with a previous study demonstrating the down-regulation of OmpW after exposure to 0.1 mg/ml of TiO_2_ in the absence of light^59^. But UV illumination is probably required to impair membrane proteins^60^,^61^ and proteins such as OmpA, OmpC and OmpF were shown to down-regulate only in the presence of UV irradiation with 0.1 mg/ml TiO_2_^59^.

**Figure 7:**
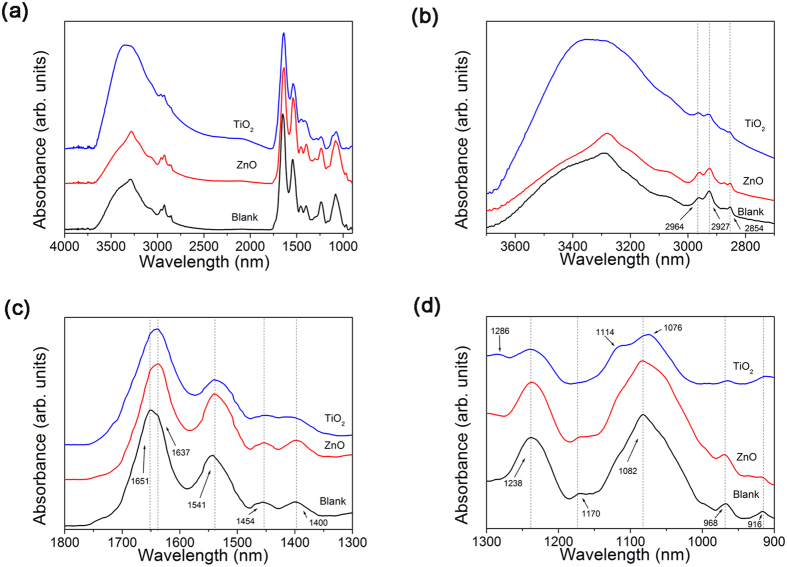
ATR-FTIR spectra of *E. coli* exposed to ZnO and TiO_2_ (**a**) entire spectral range; (**b**,**c**,**d**) relevant spectral ranges where significant changes are observed.

Difference in the response of *E. coli* bacteria to exposure to ZnO and TiO_2_ can also be observed in the ATR-FTIR spectra of the bacteria, shown in Fig. 7. Consistent with low toxicity of ZnO at higher bacteria concentrations, there is only a small difference between control and ZnO-exposed samples. For TiO_2_, we can observe a significant change in the shape of the peak corresponding to various OH group vibrations in the region 3000–3500 cm^−1 ^24,62. Furthermore, the peaks corresponding to CH_2_ and CH_3_ vibrations (four peaks in the region ~2850–2970 cm^−1^)^24^,^26^,^63^ are less pronounced. Other changes are observed in the regions corresponding to amide I, amide II and amide III bands^24^,^26^,^63^ (change in the peak shape, appearance of a new peak in samples exposed to TiO_2_ in amide III band), and finally we can also observe a significant change in the peak shape in the region 1050–1150 cm^−1^ corresponding to PO^2−^ and C-O vibrations^24^,^26^. While the possibility that the change in the shape of the OH group vibrations occurred due to the presence of TiO_2_ nanoparticles cannot be excluded, this cannot explain a reduction in the CH_2_ and CH_3_ vibrations and changes in the amide I, amide II and amide III bands, since these regions contain peaks absent in the FTIR spectra of TiO_2_ nanoparticles (see Fig. S8). The observed multiple changes in the FTIR peaks corresponding to different molecular vibrations for TiO_2_-exposed bacteria are in agreement with the proteomics data, which indicate significant changes in the membrane protein expression upon exposure to TiO_2_.

Thus, we can conclude that TiO_2_ likely interacts with LPS and/or cell membrane proteins. The nanoparticle attachment is necessary for the antibacterial action, in agreement with the literature^8^,^10^,^28^,^36^, and the toxicity under UV illumination can occur by direct charge transfer between the nanoparticles and cell membrane molecules and by mechanical disruption of the cell membrane. Although ZnO is capable of interacting with LPS molecules, the attachment between nanoparticles and LPS is likely different compared to TiO_2_ (different LPS vibration modes obtained in FTIR spectra after exposure of ZnO and TiO_2_ to LPS, Fig. S8). Compared to TiO_2_, fewer ZnO nanoparticles are attached to bacteria cells in SEM images, and consequently lower toxicity is observed despite higher ROS production and detectable response to oxidative stress in proteomics data. Significant antibacterial activity of ZnO occurs only for high ZnO concentration, where positive zeta potential values are determined and thus electrostatic interaction between bacteria membranes and ZnO nanoparticles is expected. Furthermore, some antibacterial activity also occurs in the dark for both materials for low bacterial concentrations. However, since no strong differences in antibacterial activity without illumination occur at a bacterial concentration of 10^8^ CFU/ml, no proteomic investigation was conducted for samples exposed to nanoparticles without illumination. Since neither Zn^2+^ release nor abiotic ROS production could explain antibacterial activity without illumination, this also likely occurs due to interaction between the cell membranes and nanoparticles. One possible explanation for the differences observed under illumination and in the dark is due to the differences in surface adsorbates/surface defects in ZnO and TiO_2_ under illumination which would affect the interaction with bacteria cell (in addition to any changes in the ROS generated induced by illumination). The surface defects and/or adsorbates on ZnO and TiO_2_ are affected by UV illumination, resulting in significant changes in surface wettability^64^,^65^. However, investigation of differences in the amount of adsorbed particles with and without illumination is not straightforward and requires further study.

## Conclusions

Comprehensive investigation of antibacterial activity of ZnO and TiO_2_, which are two photocatalytic materials with a similar bandgap, was performed using *E. coli* as a model organism. The two metal oxide materials induced significantly different response in the bacterial cells. ZnO resulted in lower attachment between nanoparticles and bacterial cells, higher ROS production and higher bacteria survival rate compared to TiO_2_ at the same concentrations. In the case of ZnO, the ROS-related proteins were up-regulated, while for TiO_2_ changes in the outer membrane protein expression were observed. The lack of antibacterial activity for high concentrations of bacteria and TiO_2_ can be attributed to high light scattering of the suspension, which would result in significant decrease of the UV illumination penetration into the suspension.

## Methods

### Materials and material characterization

ZnO (APS 20 nm, 99.5% purity) and TiO_2_ nanoparticles (anatase, APS 15 nm, 99% purity) were purchased from Nanostructured and Amorphous Materials, Inc. and used as received. Spin traps 5,5-Dimethyl-1-pyrroline N-oxide (DMPO) and 5-(diethoxyphosphoryl)-5-methyl-pyrroline N-oxide (DEPMPO) were obtained from Sigma-Aldrich Co. and EnzoBiochem, Inc., respectively. All chemicals were used without purification. No surfactants or dispersion agents were used to avoid possible artefacts.

The particles were dispersed by ultrasonication prior to experiments. The aggregation size and zeta potential of the nanoparticles in 0.9% NaCl solution were measured using Zetasizer 3000HSA from Malvern Instruments Ltd. Electron spin resonance (ESR) spectroscopy was used to study the reactive oxygen species (ROS) production of the nanoparticles. Spin trap DMPO was used for trapping OH^•^ radicals, and DEPMPO was used for trapping both OH^•^ radicals and superoxide ions. H_2_O_2_ solutions at different concentrations were used as standard samples to verify the position of peaks corresponding to OH^•^ radicals and superoxide ions^30^. Measurements were performed on a Bruker EMX EPR spectrometer. Suspension mixtures of 0.02 M DMPO (or 0.04 M DEPMPO) and nanoparticles in 0.9% NaCl solution were prepared for different nanoparticle concentrations (0.01 mg/ml, 0.1 mg/ml, and 1 mg/ml). The suspensions were exposed to UV illumination for 2 minutes, followed by immediate ESR measurement. For determining the metal ion release from the nanoparticles, nanoparticle suspensions in 0.9% NaCl solution were prepared and illuminated with UV light for 20 min (the same condition as in antibacterial activity experiments). The nanoparticles were removed by centrifugation and filtering. The Zn ion content in the solutions was analyzed by inductively coupled plasma mass spectroscopy (ICP-MS) with EG020F (USEPA 6020) standard.

### Antibacterial activity experiments

Gram negative bacterium *E. coli* XL1-Blue (Stratagene, USA) was used for the antibacterial activity tests. The bacteria were cultured with Luria-Bertani broth (Affymetrix USB) at 37 °C, and washed and suspended in 0.9% NaCl solution before use^30^. Bacteria and nanoparticles were mixed in a suspension inside a Petri dish to obtain different combinations of bacteria and nanoparticle concentrations. The suspension mixture was then subjected to UV illumination (365 nm, Blak-Ray^®^ B-100 AP Lamp, ~40 mW/cm^2^ measured by an optical power meter) for 20 minutes with constant stirring using a magnetic stirrer and placed in a water bath to maintain constant temperature and prevent heating due to illumination. The teflon-coated magnetic stirrer had comparable length to the diameter of Petri dish (60 mm) in which exposure experiment was conducted to prevent settlement of the nanoparticles. For the experiments in the dark, the samples were covered with an aluminium foil. The control samples were samples not exposed to nanoparticles. For all samples, serial dilution was performed and the dilution was then pipetted onto culture agar plates in triplicate. The plates were kept at 37 °C for 16 hours and the formation of colonies was observed.

### Characterization of interaction between bacteria and nanoparticles

To study the interaction between the bacteria and nanoparticles, the bacterial cells were examined by scanning electron microscopy (SEM). Cell suspensions were fixed with 2.5% glutaraldehyde in cacodylate buffer (pH 7.4) at 4 °C overnight. The cells were cleaned with cacodylate buffer and collected on a membrane (Millipore, pore size 0.8 μm). The cells were serially dehydrated and dried by critical point drying. A thin layer of Au was coated on the specimen by sputtering before examination with a LEO 1530 FEG SEM or Hitachi S4800 FEG SEM. Detailed description of proteomic analysis is given in the Supplementary Information.

### Lipid peroxidation (TBARS assay), FTIR and ATR-FTIR measurements

To examine the lipid peroxidation in cell membranes of bacteria exposed to nanoparticles, TBARS assay was performed. Bacterial cells were exposed to nanoparticles in the same way as in the antibacterial activity experiments. The cell proteins were precipitated by adding 6% w/v trichloroacetic acid (TCA) to the suspensions, incubated at room temperature for 30 minutes and then centrifuged at 11,000 g for 30 minutes. To perform the assay, 1% aqueous TBA (Sigma-Aldrich, >98%) solution was added to the supernatant in a ratio of 1:1. The mixture was boiled for 30 minutes and cooled to room temperature overnight. The absorption spectra of the solutions were measured using a UV-Vis spectrometer (PerkinElmer Lambda Bio 40).

The interaction between bacteria and nanoparticles was also studied by investigating the attachment of lipopolysaccharide (LPS) to the nanoparticles using FTIR measurements. LPS is present in the outer membrane of gram-negative bacteria, such as *E. coli*^26^. LPS from *E. coli* K-235 was obtained from Sigma-Aldrich. Nanoparticles were added into LPS aqueous solution (1 mg/ml for both nanoparticles and LPS) and dispersed by sonication. The suspension mixtures were left in ambient for 2 hours. The nanoparticles were collected by centrifugation, rinsed with de-ionized water and then dried in ambient overnight. The nanoparticles were mixed with KBr powder (infrared grade, Sigma-Aldrich) and pellets of the mixture were made. The measurement was performed on the pellets using PerkinElmer Spectrum Two IR spectrometer.

The phosphate binding properties of the nanoparticles were also examined. The nanoparticles were pre-saturated with phosphate as reported previously^24^. Nanoparticles (1 mg/ml) were immersed in an aqueous solution of Na_2_HPO_4_ (2 mg/ml) for 24 hours, followed by rinsing in de-ionized water, and drying in ambient. FTIR measurement was then performed as described. To study the effects of phosphate binding of the nanoparticles to the antibacterial properties, nanoparticles pre-saturated with phosphate and phosphate containing 0.9% NaCl solution were used. Phosphate containing 0.9% NaCl solution was prepared by the addition of a solution mixture of Na_2_HPO_4_ (24 mg/ml) and KH_2_PO_4_ (5.7 mg/ml) into NaCl solution to obtain a solution with phosphate concentration of ~50 μg/ml. Antibacterial experiments were performed as described previously. For ATR-FTIR measurements, nanoparticles were dispersed in a 0.9% w/v NaCl solution at a concentration 2 mg/ml. Then, 1 ml of a bacteria suspension in 0.9% NaCl was added. The final concentration of the bacteria in the suspension exhibited OD 2.0. The samples were then illuminated with UV light. The bacterial cells were collected by centrifugation at 9000 rpm for 1 min. followed by discarding the supernatants, re-suspending the cells in a 0.9% NaCl solution and repeating the centrifugation procedure one more time. The cell suspensions were then drop-cast on double-side polished Si substrates and dried at room temperature overnight. The ATR-FTIR measurements were performed using a Bruker Vertex 70 FTIR spectrometer.

## Additional Information

**How to cite this article**: Leung, Y. H. *et al*. Toxicity of ZnO and TiO_2_ to *Escherichia coli* cells. *Sci. Rep.* **6**, 35243; doi: 10.1038/srep35243 (2016).

## Supplementary Material

Supplementary Information

## Figures and Tables

**Table 1:** Nanoparticle properties in 0.9% NaCl solution for different concentrations.

Sample	C	Aggregation size (nm)	Zeta potential (mV)	Zn^2+^(ppb)	Turbidity (FTU)
ZnO	0.01	166(33%) 336(67%)	−21.5	379	10.9
	0.1	235(76%) 759(24%)	−11.8	510	155
	1.0	1377(100%)	8.8	720	>1000
TiO_2_	0.01	221 (12%) 610(88%)	−24.6	—	49.5
	0.1	583(100%)	−19.3	—	711
	1.0	334(100%)	N/A	—	>1000

Zn^2+^ concentration in the control sample was below the detection limit (10 ppb). C denotes sample concentration in mg/ml, FTU denotes formazin turbidity unit. N/A denotes not measurable value.
