# Supplementary material for: Toxicity of ZnO and TiO2 to Escherichia coli cells
Source: Sci Rep. 2016 Oct 12;6:35243. doi: 10.1038/srep35243 (PMC5378928; doi:10.1038/srep35243)
Supplement: Supplementary Information [file srep35243-s1.doc]

**Supplementary Information**

Toxicity of ZnO and TiO_2_ to *Escherichia coli* cells

Yu Hang Leung,^a^ Xiaoying Xu,^b^ Angel P. Y. Ma,^c^ Fangzhou Liu,^a^ Alan M. C. Ng,^d,a^ Zhiyong Shen,^b^ Lee A. Gethings,^e^ Mu Yao Guo,^a^ Aleksandra B. Djurišić,^a^* Patrick K. H. Lee,^b^* Hung Kay Lee,^f^ Wai Kin Chan,^g^ and Frederick C. C. Leung^c^

Table S1. Number of *E. coli* bacteria colonies formed on agar plates for bacteria exposed to ZnO nanoparticles of different concentrations in the dark (D) and with 20 minutes of UV illumination (L). SD denotes standard deviation.

| Initial Bacteria Concentration (CFU/ml) | Nanoparticle Concentration (mg/ml) | Colonies counts | Mean ± SD | Survival Rate (%) |
| --- | --- | --- | --- | --- |
| 10^6^ | 0 | 848, 1204, 1102 (L)  1128,1480,1576 (D) | 1051 ± 183 (L)  1395± 236 (D) | 100.0 (L)  100.0 (D) |
|  | 0.01 | 884, 892, 926 (L)  776, 768, 880 (D) | 901 ± 22 (L)  808± 62 (D) | 85.7 (L)  57.9 (D) |
|  | 0.1 | 98, 85, 123 (L)  168, 184, 356 (D) | 102 ± 19 (L)  236± 104 (D) | 9.7 (L)  16.9 (D) |
|  | 1 | 0, 0, 0 (L)  0, 3, 2 (D) | 0 ± 0 (L)  2± 2 (D) | 0.0 (L)  0.1 (D) |
| 10^7^ | 0 | 1336, 1064, 1240 (L)  1902, 2328, 2118 (D) | 1213 ±138 (L)  2116± 213 (D) | 100.0 (L)  100.0 (D) |
|  | 0.01 | 980, 1120, 904 (L)  720, 1400, 976 (D) | 1001 ± 110 (L)  1032± 343 (D) | 82.5 (L)  48.7 (D) |
|  | 0.1 | 1120, 786, 816 (L)  436, 464, 504 (D) | 907 ± 185 (L)  468± 34 (D) | 74.8 (L)  22.1 (D) |
|  | 1 | 5, 8, 5 (L)  112, 240, 240 (D) | 6 ± 2 (L)  197± 74 (D) | 0.5 (L)  9.3 (D) |
| 10^8^ | 0 | 501, 541, 441 (L)  840, 1104, 1260 (D) | 494 ± 50 (L)  1068± 212 (D) | 100.0 (L)  100.0 (D) |
|  | 0.01 | 534, 676, 720 (L)  776, 840, 976 (D) | 643 ± 97 (L)  864± 102 (D) | 130.1 (L)  71.6 (D) |
|  | 0.1 | 626, 520, 532 (L)  1008, 860, 832(D) | 559 ± 58 (L)  900± 95 (D) | 113.1 (L)  74.6 (D) |
|  | 1 | 236, 175, 246 (L)  1146, 960, 852 (D) | 219 ± 38 (L)  986± 149 (D) | 44.3 (L)  81.8 (D) |

Table S2**.** Number of *E. coli* bacteria colonies formed on agar plates for bacteria exposed to TiO_2_ nanoparticles of different concentrations in the dark (D) and with 20 minutes of UV illumination (L). SD denotes standard deviation.

| Initial Bacteria Concentration (CFU/ml) | Nanoparticle Concentration (mg/ml) | Colonies counts | Mean ± SD | Survival Rate (%) |
| --- | --- | --- | --- | --- |
| 10^6^ | 0 | 868, 874, 912 (L)  1170,1956, 1884 (D) | 885 ± 24 (L)  1670± 435 (D) | 100.0 (L)  100.0 (D) |
|  | 0.01 | 66, 51, 33 (L)  618, 582, 918 (D) | 50 ± 17 (L)  706± 184 (D) | 5.7 (L)  42.1 (D) |
|  | 0.1 | 22, 3, 5 (L)  181, 192, 140 (D) | 10 ± 10 (L)  171± 27(D) | 1.1 (L)  10.3 (D) |
|  | 1 | 0, 0, 0 (L)  86, 95, 39 (D) | 0 ± 0 (L)  73± 30 (D) | 0.0 (L)  4.4 (D) |
| 10^7^ | 0 | 1382, 1196, 1232 (L)  1902, 2328, 2118 (D) | 1270 ± 99 (L)  2116± 213(D) | 100.0 (L)  100.0 (D) |
|  | 0.01 | 99, 102, 93 (L)  1020, 1176, 786 (D) | 98 ± 5 (L)  994± 196 (D) | 7.7 (L)  47.0 (D) |
|  | 0.1 | 111, 140, 130 (L)  1368, 1452, 522 (D) | 127 ± 15 (L)  1114± 514 (D) | 10.0 (L)  52.7 (D) |
|  | 1 | 33, 27, 7 (L)  552, 816, 630 (D) | 22 ± 14 (L)  666± 136 (D) | 1.8 (L)  31.4 (D) |
| 10^8^ | 0 | 1123, 1267, 1038 (L)  840, 1104, 1260 (D) | 1143 ± 116 (L)  1068± 212 (D) | 100.0 (L)  100.0 (D) |
|  | 0.01 | 908, 796, 840 (L)  558, 1164, 834 (D) | 848 ± 56 (L)  852± 303 (D) | 74.2 (L)  70.6 (D) |
|  | 0.1 | 81, 75, 83 (L)  1128, 948, 948 (D) | 80 ± 4 (L)  1008± 104 (D) | 7.0 (L)  83.6 (D) |
|  | 1 | 1423, 1256, 1300 (L)  1098, 1152, 798 (D) | 1326 ± 87 (L)  1014± 190 (D) | 116.1 (L)  84.1 (D) |


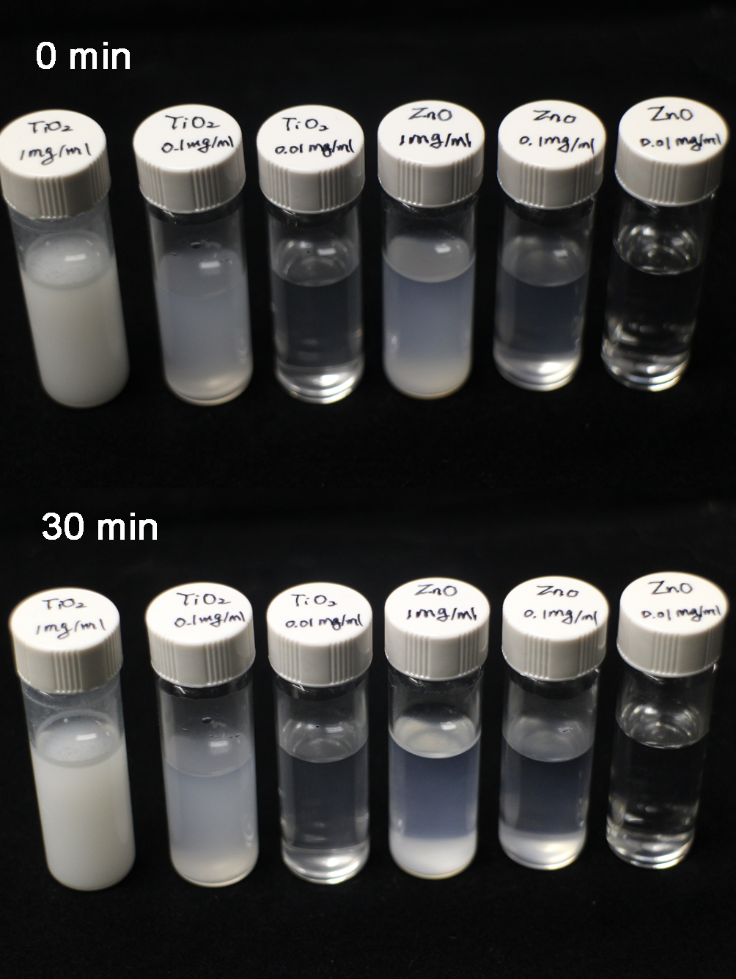


Figure S1. Photos of nanoparticle suspensions at different concentrations (immediately after dispersion and after 30 min.).


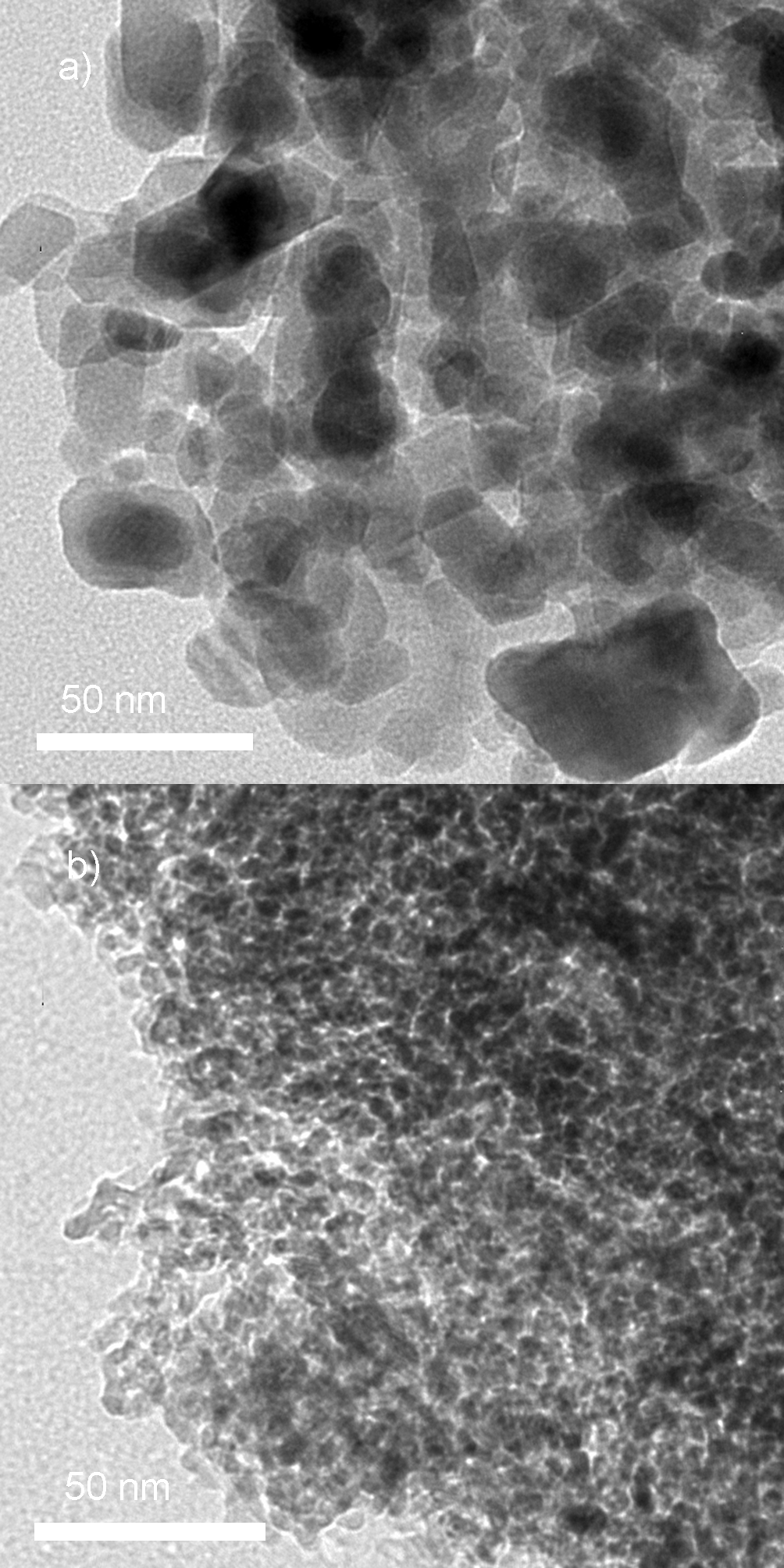


Figure S2. TEM images of a) ZnO and b) TiO_2_ nanoparticles.


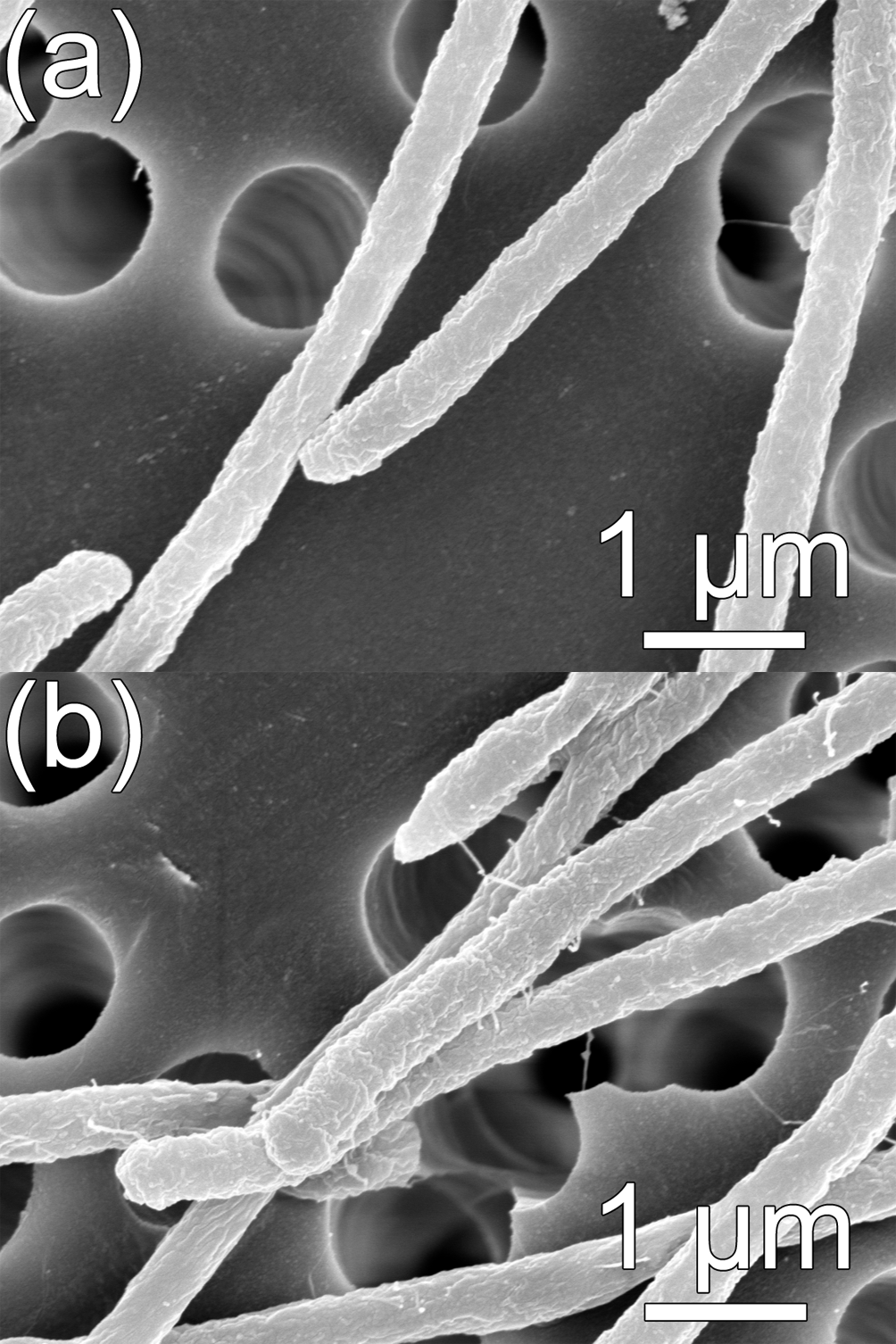


Figure S3. Representative SEM images of *E. coli* bacterial cells without the exposure to nanoparticles, a) in 0.9% NaCl and b) in 0.9% NaCl with 50 µg/ml phosphate.

**
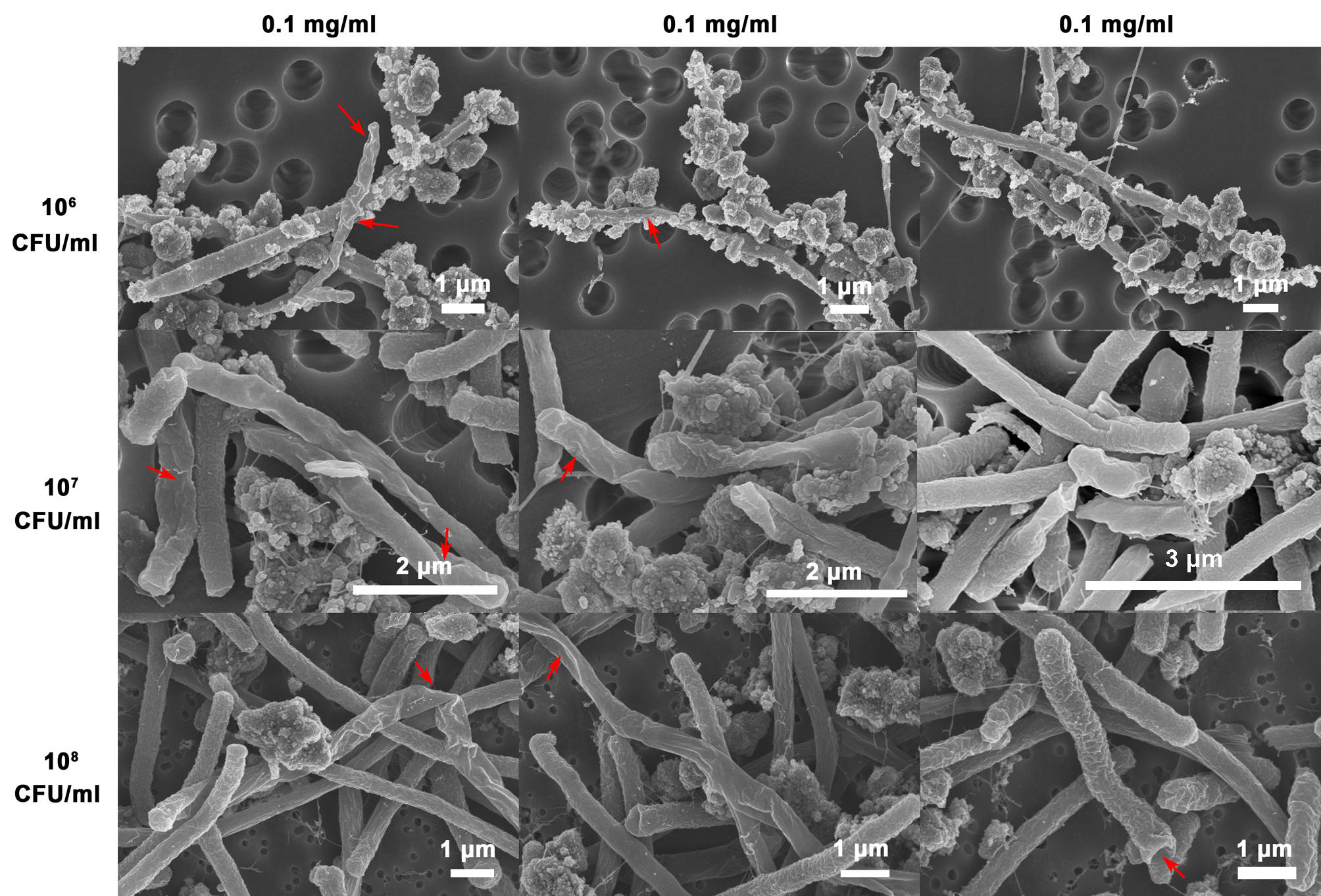
**

Figure S4. Representative SEM images of *E. coli* bacterial cells exposed to TiO_2_ nanoparticles at a concentration 0.1 mg/ml.

**
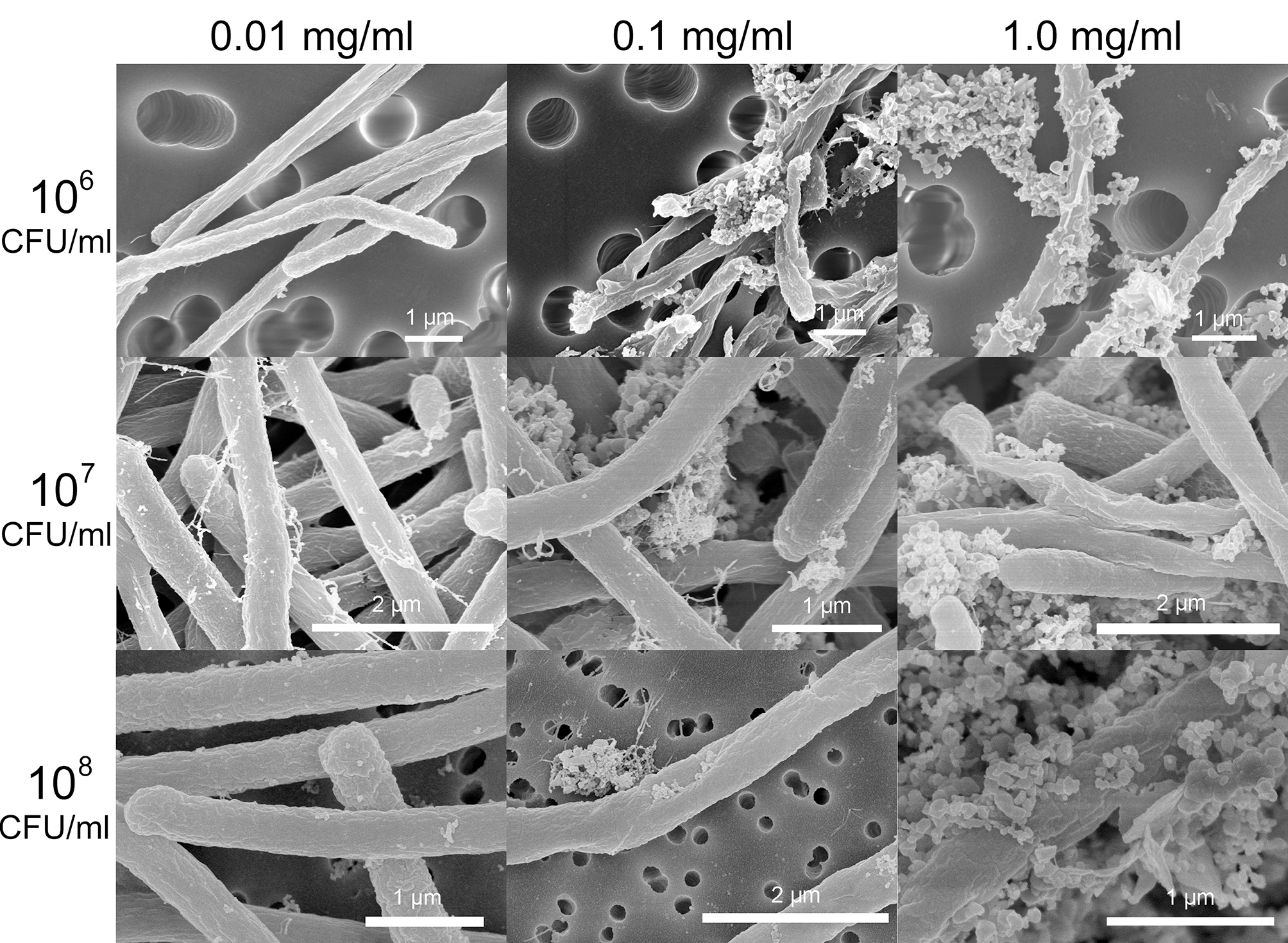
**

Figure S5. Representative SEM images of *E. coli* bacterial cells after exposure to ZnO nanoparticles with different concentrations (0.01, 0.1 and 1.0 mg/ml). Left-most column indicates the initial bacterial concentrations (10^6^, 10^7^, and 10^8^ CFU/ml).


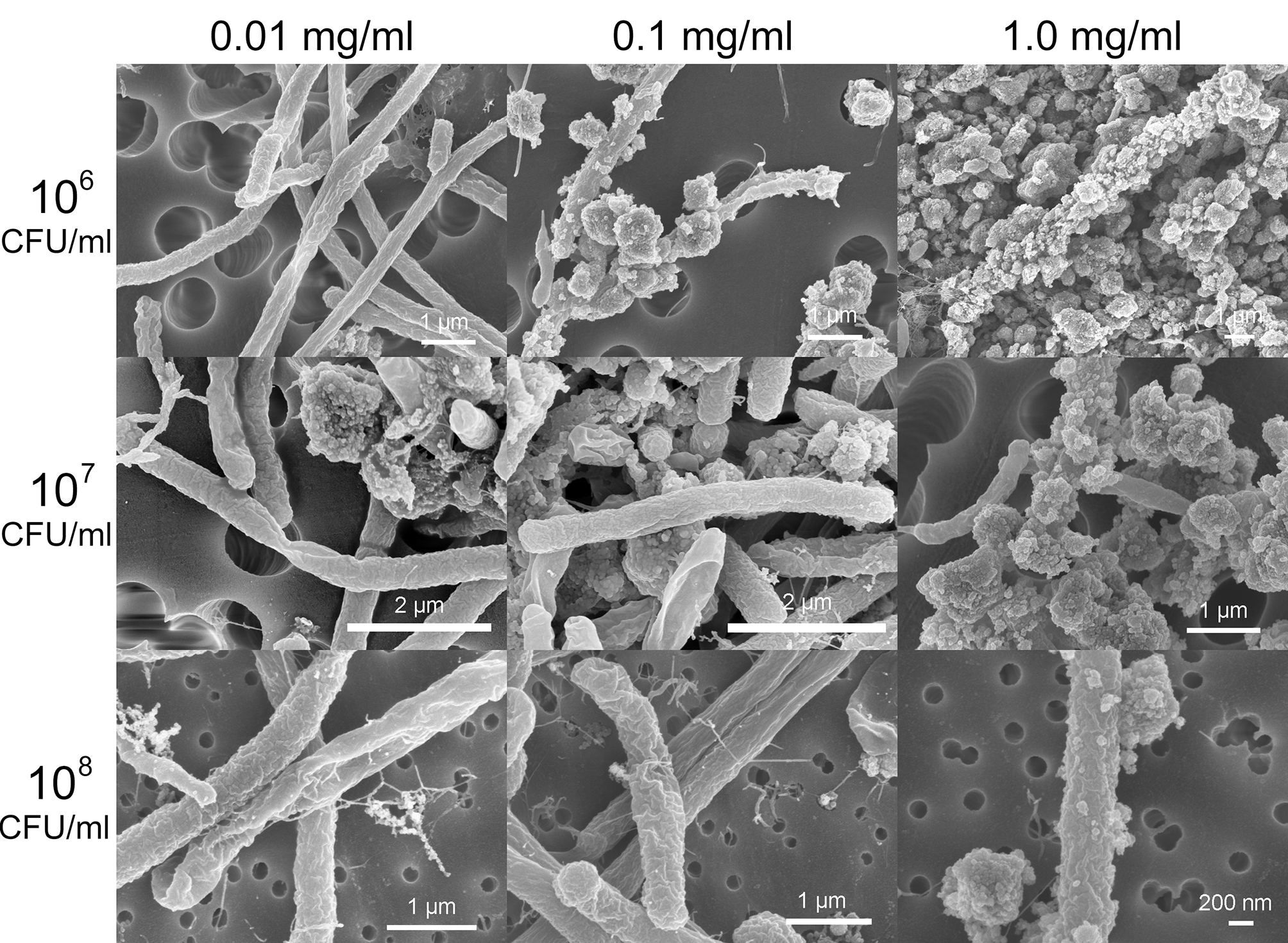


Figure S6. Representative SEM images of *E. coli* bacterial cells after exposure to TiO_2_ nanoparticles with different concentrations (0.01, 0.1 and 1.0 mg/ml). Left-most column indicates the initial bacterial concentrations (10^6^, 10^7^, and 10^8^ CFU/ml).

Table S3. TBARS assay on *E. coli* exposed to ZnO and TiO_2_ nanoparticles.

| Bacteria concentration (CFU/ml) | Sample | Sample concentration (mg/ml) | Absorption@532 nm (×10^-3^) |
| --- | --- | --- | --- |
| 0 | Control | N/A | 4.0 |
|  | ZnO | 0.01 | 2.9 |
|  |  | 0.1 | 2.6 |
|  |  | 1.0 | 1.1 |
|  | TiO_2_ | 0.01 | 2.8 |
|  |  | 0.1 | 1.1 |
|  |  | 1.0 | 1.1 |
| 10^6^ | Control | N/A | 1.9 |
|  | ZnO | 0.01 | 3.2 |
|  |  | 0.1 | 4.9 |
|  |  | 1.0 | 2.4 |
|  | TiO_2_ | 0.01 | 1.5 |
|  |  | 0.1 | 1.9 |
|  |  | 1.0 | 2.7 |
| 10^7^ | Control | N/A | 5.8 |
|  | ZnO | 0.01 | 4.5 |
|  |  | 0.1 | 13.9 |
|  |  | 1.0 | 29.6 |
|  | TiO_2_ | 0.01 | 5.4 |
|  |  | 0.1 | 2.9 |
|  |  | 1.0 | 6.3 |
| 10^8^ | Control | N/A | 1.8 |
|  | ZnO | 0.01 | 6.4 |
|  |  | 0.1 | 5.39 |
|  |  | 1.0 | 9.6 |
|  | TiO_2_ | 0.01 | 2.6 |
|  |  | 0.1 | 12.6 |
|  |  | 1.0 | 2.9 |

Table S4. Number of *E. coli* bacteria colonies formed on agar plates for bacteria exposed to 0.9% NaCl solution containing 720 ppb of Zn^2+^ ions with 20 minutes of UV illumination. SD denotes standard deviation.

| Initial Bacteria Concentration (CFU/ml) | Medium | Colonies counts | Mean ± SD | Survival Rate (%) |
| --- | --- | --- | --- | --- |
| 10^6^ | NaCl | 672, 620, 626 | 639 ± 28 | 100.0 |
|  | NaCl + Zn^2+^ | 570, 440, 590 | 533 ± 81 | 83.4 |
| 10^7^ | NaCl | 748, 598, 750 | 699 ± 87 | 100.0 |
|  | NaCl + Zn^2+^ | 728, 488, 826 | 681 ± 174 | 97.4 |
| 10^8^ | NaCl | 834, 660, 810 | 768 ± 94 | 100.0 |
|  | NaCl + Zn^2+^ | 974, 726, 772 | 824 ± 132 | 107.3 |

Table S5. Number of *E. coli* bacteria colonies formed on agar plates for bacteria exposed to nanoparticles with and without phosphate pre-treatment. 0.9% NaCl media with and without the addition of phosphate were used. UV illumination time was 20 minutes. Nanoparticle concentration was 0.1 mg/ml and initial bacteria concentration is 10^6^ CFU/ml. SD denotes standard deviation.

| Medium | Sample | Colonies counts | Mean ± SD | Survival Rate (%) |
| --- | --- | --- | --- | --- |
| 0.9% NaCl | Control | 552, 704, 662 | 639 ± 78 | 100.0 |
|  | ZnO | 16, 17, 22 | 18 ± 3 | 2.9 |
|  | TiO_2_ | 1, 8, 5 | 5 ± 4 | 0.7 |
|  | ZnO-P | 106, 110, 94 | 103 ± 8 | 16.2 |
|  | TiO_2_-P | 42, 44, 36 | 41 ± 4 | 6.4 |
| 0.9% NaCl +50 μg/ml phosphate | Control | 499, 288, 402 | 396 ± 106 | 100.0 |
|  | ZnO | 630, 628, 606 | 621 ± 13 | 156.8 |
|  | TiO_2_ | 26, 25, 20 | 24 ± 3 | 6.0 |


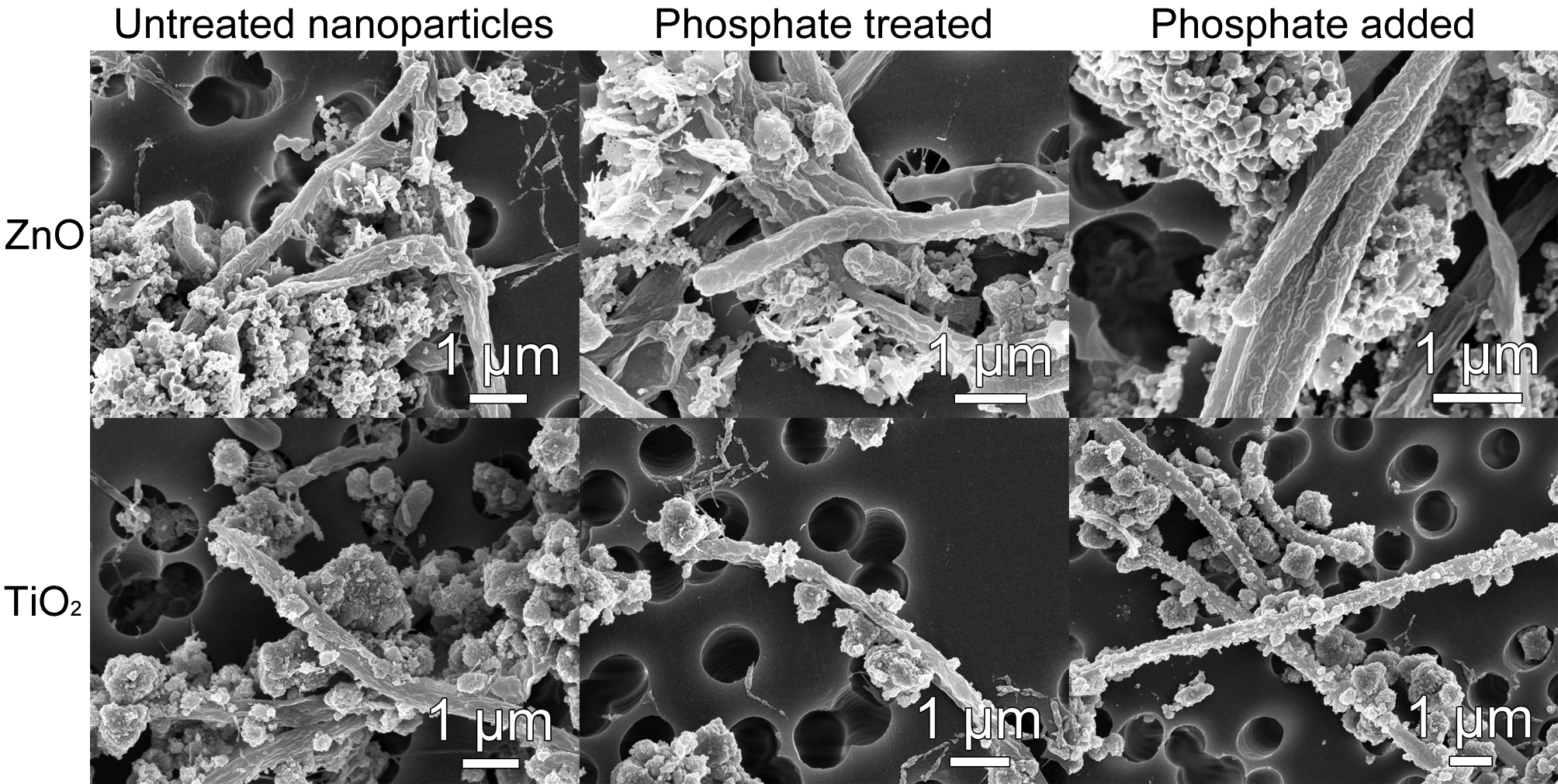


Figure S7. Representative SEM images of *E. coli* bacterial cells after exposure to untreated nanoparticles (left column), phosphate treated nanoparticles (middle column), and untreated nanoparticles with the addition of phosphate in the suspension (right column). Nanoparticle concentration and initial bacterial concentration are 0.1 mg/ml and 10^6^ CFU/ml, respectively.


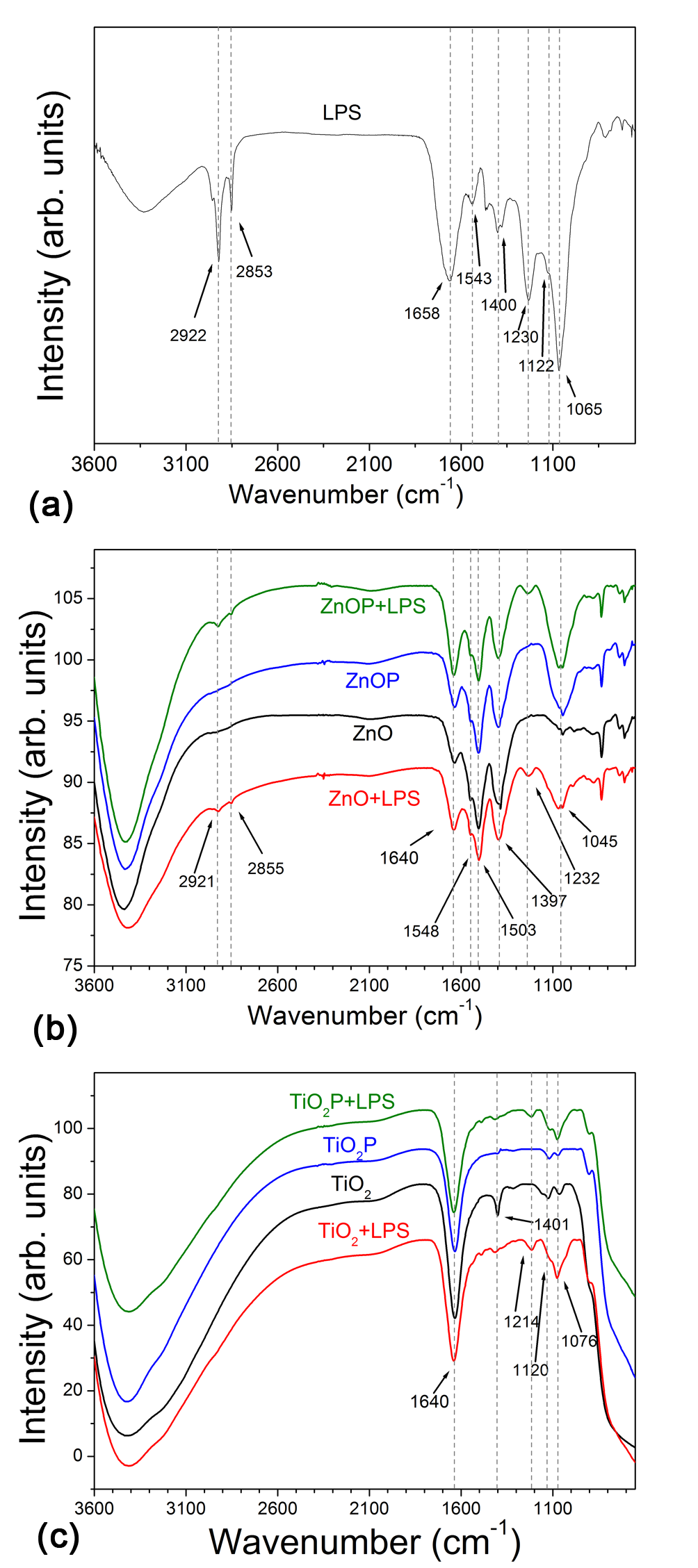


Figure S8. FTIR spectra of (a) LPS only, (b) ZnO nanoparticles and (c) TiO_2_ nanoparticles with and without phosphate treatment and LPS exposure. The spectra have been vertically shifted for clarity.

Table S6. Number of *E. coli* bacteria colonies formed on agar plates for bacteria exposed to 20 minutes of UV illumination with and without the addition of H_2_O_2_. Initial bacteria concentration was 10^8^ CFU/ml. SD denotes standard deviation.

| Sample | Colonies counts | Mean ± SD | Survival Rate (%) |
| --- | --- | --- | --- |
| 0% H_2_O_2_ | 204, 126, 176 | 169 ± 40 | 100.0 |
| 0.01% H_2_O_2_ | 52, 43, 47 | 47 ± 5 | 27.8 |

Table S7 Number of *E. coli* bacteria colonies formed on agar plates for bacteria exposed to 0 °C/55 °C thermal stress treatment with different periods of time. Initial bacteria concentration was 10^8^ CFU/ml. SD denotes standard deviation.

| Treatment time  (min.) | Colonies counts | Mean ± SD | Survival Rate (%) |
| --- | --- | --- | --- |
| 0 | 475, 402, 335 | 404 ± 70 | 100.0 |
| 1 | 204, 270, 272 | 249 ± 39 | 61.6 |
| 2 | 178, 185, 224 | 196 ± 25 | 48.5 |
| 5 | 1, 5, 5 | 4 ± 2 | 1.0 |


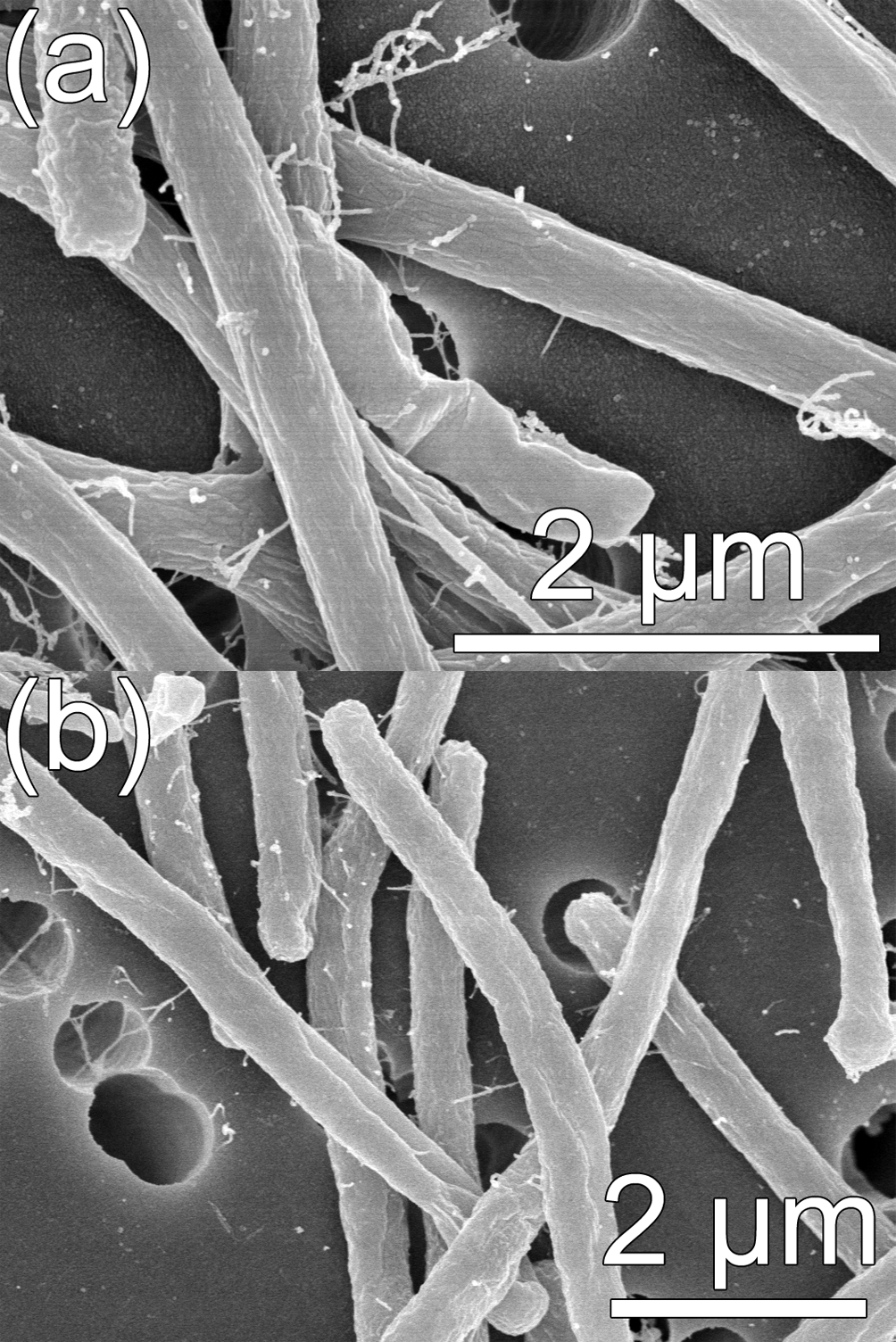


Figure S9. Representative SEM images of *E. coli* bacterial cells exposed to a) 0.01% H_2_O_2_ with 20 minutes of UV illumination, and b) 5 minutes of 0 °C/55 °C thermal stress treatment.

Table S8. Expression of selected membrane proteins after incubating with TiO_2_, ZnO and H_2_O_2_ for 10, 20 and 30 minutes.

| **Accession no.** | **Description** | **Expression ratio** | | | | | | | | | | | |
| --- | --- | --- | --- | --- | --- | --- | --- | --- | --- | --- | --- | --- | --- |
|  |  | **TiO_2_ (0.1 mg/ml)** | | | **ZnO (0.1 mg/ml)** | | | **ZnO (0.01 mg/ml)** | | | **H_2_O_2_ (0.01%)** | | |
|  |  | 10 mins | 20 mins | 30 mins | 10 mins | 20 mins | 30 mins | 10 mins | 20 mins | 30  mins | 10 mins | 20 mins | 30  mins |
| **P31064** | Inner membrane protein YedE | 1.0 | -1.1 | -2.2 | 1.2 | 6.3* | 2.9 | 1.4 | 4.0 | 1.2 | 2.0 | 5.5* | 2.8 |
| **P37665** | Inner membrane lipoprotein YiaD | 0.0 | 0.0 | -3.1 | -2.4 | 6.2 | 4.3 | 3.6 | 7.3 | -20.0 | 8.8 | 36.0* | 6.4 |
| **P0ADZ7** | Membrane protein YajC | -1.5 | 1.1 | -1.0 | -1.5 | -1.7 | -1.9 | -2.0 | -1.7 | -1.3 | -3.6* | -3.7* | 3.0* |
| **P0A927** | Nucleoside-specific channel-forming protein tsx | -1.3 | 1.1 | -1.1 | 1.0 | 1.1 | -1.2 | -1.1 | 1.1 | 1.1 | -1.9 | -1.9 | -1.9 |
| **P0A910** | Outer membrane protein A (OmpA) | -1.3 | 1.1 | -1.0 | -2.3* | -2.7* | -3.2 | -3.3* | -2.4* | -1.7 | -3.0* | -3.2* | -2.4 |
| **P06996** | Outer membrane protein C (OmpC) | -6.7 | 1.0 | -1.1 | 1.1 | 2.3 | 1.2 | 1.2 | 1.8 | 1.2 | -1.5 | -1.1 | 1.0 |
| **P02931** | Outer membrane protein F (OmpF) | -2.1 | 1.0 | -1.0 | 1.4 | 2.3* | 1.2 | 1.0 | 1.8 | 1.4 | -1.1 | 1.0 | 1.2 |
| **P0A915** | Outer membrane protein W (OmpW) | -2.2 | -1.4 | 0.0 | 11.1 | 32.9 | 6.1 | 2.7 | 3.8* | 6.0 | 5.6* | 19.8* | 11.1* |
| **P30130** | Outer membrane usher protein FimD | 0.0 | 1.1 | 2.0 | 2.4 | 3.9 | 2.8 | 6.3 | 5.8 | 1.8 | 21.1* | 21.5* | 11.5* |
| **P02930** | Outer membrane protein TolC | -16.7 | 1.0 | -3.3* | -1.6 | 1.6 | -1.1 | -1.7 | 1.8 | -1.1 | -1.9 | 1.1 | -1.1 |
| **P0A905** | Outer membrane lipoprotein | -1.7 | 1.1 | -1.1 | -1.1 | -1.1 | -1.5 | -1.4 | -1.0 | -1.1 | -2.9* | -2.7* | -2.6 |

“*” indicates proteins that were statistically significant (FDR ≤ 1%, peptide count ≥ 3, fold-change ≥ 2)

Table S9**.** Expression of selected membrane proteins after incubating with TiO_2_ (1 mg/ml) and ZnO (1 mg/ml) for 10, 20 and 30 minutes.^a^

| **Accession no.** | **Description** | **Expression ratio** | | | | | |
| --- | --- | --- | --- | --- | --- | --- | --- |
|  |  | **TiO_2_ (1 mg/ml)** | | | **ZnO (1 mg/ml)** | | |
|  |  | 10 mins | 20 mins | 30 mins | 10 mins | 20 mins | 30 mins |
| P31064 | Inner membrane protein YedE | 1.0 | 1.1 | -1.2 | -1.1 | -1.1 | 1.0 |
| P77307 | Inner membrane protein YbbM | 1.1 | -1.2 | 1.4 | 1.2 | -1.5 | 1.6 |
| P37665 | Inner membrane lipoprotein YiaD | -1.2 | -1.4 | -3.1 | -1.5 | -1.5 | -2.1 |
| P0ADZ7 | Membrane protein YajC | -1.0 | -1.1 | 1.1 | 1.0 | -1.2 | 1.1 |
| P0A927 | Nucleoside-specific channel-forming protein tsx | -1.0 | -1.1 | 1.1 | 1.1 | -1.1 | 1.4 |
| P0A910 | Outer membrane protein A (OmpA) | 1.1 | -1.0 | 1.1 | 1.2 | 1.1 | 1.2 |
| P06996 | Outer membrane protein C (OmpC) | 1.3 | 1.2 | -1.2 | 1.6 | 1.4 | 1.2 |
| P02931 | Outer membrane protein F (OmpF) | 1.1 | -1.1 | 1.3 | 2.1 | 1.2 | 2.3 |
| P0A915 | Outer membrane protein W (OmpW) | 1.1 | 1.1 | -1.1 | 1.2 | -1.1 | 1.2 |
| P30130 | Outer membrane usher protein FimD | 1.1 | -1.0 | 1.3 | 1.2 | -1.0 | 1.4 |
| P02930 | Outer membrane protein TolC | 1.3 | 1.0 | 1.3 | 1.4 | 1.1 | 1.5 |
| P0A905 | Outer membrane lipoprotein | 1.2 | 1.2 | 1.0 | 1.8 | 1.6 | 1.4 |
| P77339 | Uncharacterized lipoprotein | -1.3 | 1.3 | 1.4 | -1.2 | 1.3 | 1.5 |

^a^ None of the proteins listed here was statistically significant.

**Experimental Details**

***Proteomic analysis:*** Bacterial cell samples for proteomic analysis were prepared with a similar method as in antibacterial activity experiments. Bacteria and nanoparticles with different concentrations were mixed in a suspension. The overall bacteria concentration was 10^8^ CFU/ml. The suspension was subjected to UV illumination (365 nm, Blak-Ray® B-100 AP Lamp, ~40 mW/cm^2^) for different periods of time. Bacterial cells were then collected by centrifugation and the supernatant was discarded. The resultant cell pellets were kept at -80 °C before further processing. Triplicate samples were analyzed for each condition by mass spectrometry (MS) as previously described.^24^ Briefly, proteins were digested by sequencing grade Thymidylate kinase-treated trypsin and 100 ng/µl of sample were used for 1D liquid chromatography (LC)-MS analysis. 1D nanoscale LC separation of tryptic peptides was performed with a nanoAcquity system (Waters Corporation, Milford, MA) and mass spectrometric analysis was conducted using a Synapt G2-S mass spectrometer (Waters Corporation, Manchester, UK) using settings as previously described.^24^ The TransOmics Informatics (Waters Corporation, Manchester, UK) software was used to process the acquired data. Protein identification was based on a search against *E. coli* (strain K12) in the UniProt database. Statistically significant proteins were determined using R scripts by a two-tailed *t-*test with correction to control the false-discovery rate (FDR) to ≤ 1% as described previously.^24^ A statistical significant protein must satisfy the following: FDR ≤ 1%, peptide count ≥ 3, and fold-change ≥ 2. In this study, up-regulation (positive expression ratios) refers to a higher abundance of protein in the samples treated with nanomaterials, H_2_O_2_ or thermal stress relative to the control without treatment, while down-regulation (negative expression ratios) refers to fewer proteins in the treated samples.

***Zn^2+^ ion toxicity, H_2_O_2_ and thermal stress experiments, phosphate binding properties determination:***To study the effects of Zn^2+^ ions to the viability of the *E. coli* bacteria, antibacterial experiments were performed with 0.9% NaCl solution with and without the addition of Zn^2+^ ions. Anhydrous zinc acetate (99.99% purity, Sigma Aldrich) was used as the Zn^2+^ ions source. The overall concentration of Zn^2+^ ions is ~720 ppb. For the antibacterial testing with hydrogen peroxide (H_2_O_2_), the *E. coli* bacteria were mixed with 0.9% NaCl solution with 0.01% H_2_O_2_. The initial overall bacterial concentration was 10^8^ CFU/ml. The suspension mixture was then subjected to UV illumination for 20 minutes with constant stirring. Serial dilution was performed and the dilution was then pipetted onto culture agar plates in triplicate. The plates were kept at 37 °C for 16 hours and the formation of colonies was observed. For the thermal stress treatment on bacterial cells, the *E. coli* bacterial cells (10^8^ CFU/ml) were put into an ice bath and consequently to a water bath heated to 55 °C for different periods of time (1, 2 or 5 minutes). The viability of the cells was determined by observing the formation of colonies.
